# Surgical and multitreatment approach in a case of primary cardiac angiosarcoma: A case report

**DOI:** 10.1016/j.ijscr.2022.107349

**Published:** 2022-06-24

**Authors:** Akihiro Kobayashi, Yoshimori Araki, Takafumi Terada, Sachie Terazawa, Osamu Kawaguchi

**Affiliations:** a Department of Cardiac Surgery, Toyota Kosei Hospital, Ibobara 500-1, Jousui-cho, Toyota, Aichi 470-0396, Japan; b Department of Cardiac Surgery, Nagoya University Hospital, 65 Tsurumai-cho, Showa-ku, Nagoya, Aichi 466-8550, Japan

**Keywords:** Angiosarcoma, Cardiac tumor, Surgery, Chemotherapy, Radiation, Pericardial effusion

## Abstract

**Introduction and importance:**

Primary cardiac angiosarcoma is extremely rare, and its prognosis remains poor, with a mean life expectancy of only a few months. Here, we report a case of primary cardiac angiosarcoma.

**Case presentation:**

A 49-year-old Japanese woman with a month-long history of dyspnea was admitted to our hospital for pericardial effusion. Chest computed tomography and cardiac magnetic resonance imaging showed a mass in the right atrium. The patient underwent surgical resection of the tumor, and the pathological diagnosis was angiosarcoma. The patient received radiotherapy after surgery. Six months following surgery, she underwent chemotherapy following the diagnosis of lung metastasis. The patient died 18 months after the initial diagnosis.

**Clinical discussion:**

Cardiac angiosarcoma is rare and difficult to diagnose early because it is associated with few symptoms. Moreover, there are currently no established guidelines for the treatment of this disease because of its rarity and sparse descriptive literature Therefore, multidisciplinary therapies should be considered, including surgery, radiotherapy, and chemotherapy.

**Conclusion:**

There is no standard treatment for cardiac angiosarcoma, but surgical resection, chemotherapy, radiation therapy, or a combination of these therapies may be useful.

## Highlights

• Pericardial fluid cytology has little value in diagnosing cardiac angiosarcomas.
• Imaging studies are essential for the diagnosis of primary cardiac angiosarcomas.
• Surgery and radiochemotherapy extended survival to up to 18 months.

## 1 Introduction

Although primary cardiac angiosarcoma is the most common primary malignant tumor of the heart, it is extremely rare [1]. Its diagnosis is difficult and often delayed owing to its aggressive characteristics and nonspecific constitutional symptoms. Therefore, the prognosis of patients with cardiac angiosarcoma is usually poor.

We report a case of a 49-year-old woman suffering from primary cardiac angiosarcoma presenting with dyspnea and pericardial effusion. This case report is compliant with the SCARE Guidelines [2].

## 2 Case presentation

**Fig. 1:**
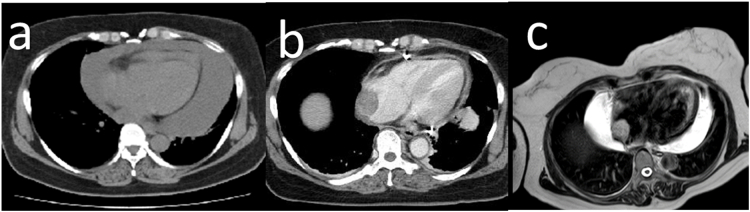
Computed tomography shows (a) large epicardial effusion and (b) tumor in the right atrium. (c) Magnetic resonance imaging shows epicardial effusion. The mass in the right atrium had an isointense to high-intensity signal on T2-weighted images.

A 49-year-old woman with a 1-month history of dyspnea was admitted to our hospital. Her medical history included hypertension, hyperlipidemia, and uterine fibroids. Chest computed tomography (CT) and echocardiography revealed a large epicardial effusion (Fig. 1A). The patient underwent pericardiocentesis to remove 1.2 L of hemorrhagic pericardial fluid. Cytology of the fluid was negative for malignant cells. Contrast-enhanced CT after pericardiocentesis showed a tumor measuring 24 × 33 mm in the right atrium (Fig. 1B). Following pericardiocentesis, the patient's symptoms were alleviated, and she was discharged. Two weeks later, transthoracic echocardiography and cardiac magnetic resonance imaging (MRI) showed progression of the epicardial effusion. Since there were no severe symptoms, we did not intervene. MRI showed a 25 × 28-mm mass in the right atrium exhibiting high signal intensity on T2-weighted images (Fig. 1C). Blood test results for tumor markers were negative. Coronary artery angiography revealed a feeding artery from the sinus node branch to the tumor. We investigated for embolization of the feeding artery, although we could not find any previous report of such embolization treatment of the feeding artery in the case of a cardiac tumor. These examinations were insufficient to make a definitive diagnosis. However, the tumor was resected to alleviate the persistent tamponade.

**Fig. 2:**
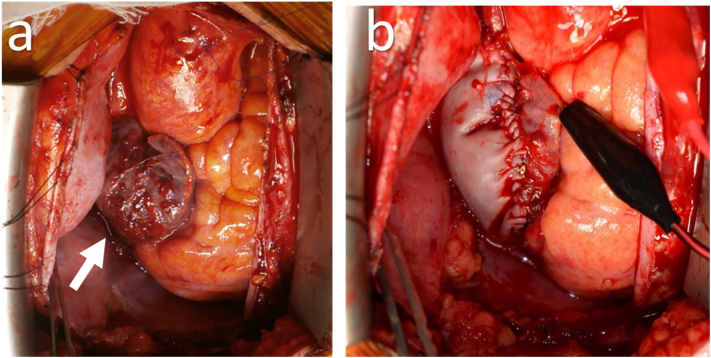
(a) Intra-operative pictures of tumor. (b) The right atrium was reconstructed with a bovine pericardial patch.

**Fig. 3:**
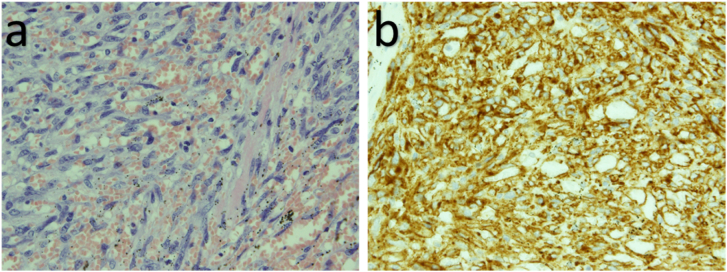
(a) Photomicrograph shows immature fusiform cells with a rich blood supply. (b) Immunohistochemistry demonstrating the tumor was CD34 positive.

**Fig. 4:**
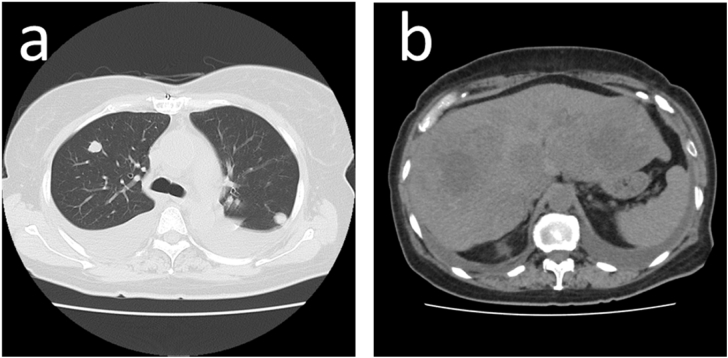
Computed tomography shows lung metastasis (a) and liver metastasis (b).

Preoperative transesophageal echocardiography, performed under general anesthesia, showed a mass measuring 28 × 41 mm originating from the free wall of the right atrium. It was not adherent to the tricuspid valve or septum and was thus resectable. Upon pericardiotomy, the tumor was visualized in the right atrium, adherent to the pericardium. The tumor was highly vascularized and had rough borders (Fig. 2). Cardiopulmonary bypass was established between the ascending aorta and superior vena cava. A venous cannula was subsequently inserted into the right femoral artery, through the inferior vena cava, and into the front of the right atrium so as not to interfere with the tumor. We made an incision on the right atrium near the tumor and observed the presence of endocardium on the smooth surface of the mass. We biopsied a part of the tumor surface to obtain an intraoperative frozen section, which revealed the presence of a sarcoma. The tumor and adherent right atrial wall were removed with adequate margins to the greatest extent possible. The resected right atrial wall was reconstructed using a bovine pericardial patch. Pathological examination of the resected tumor revealed fusiform tumor cells in frequent mitosis and a rich blood supply with intraluminal red blood cells. Immunological staining was positive for CD34 expression (Fig. 3). A pathological diagnosis of angiosarcoma was made; the resected end of the superior vena cava was tumor-free. The patient was discharged on postoperative day 12. Postoperative CT and echocardiography showed no residual tumor, while positron emission tomography revealed normal findings. Two months later, the patient received radiotherapy (heavy ion therapy) to the right atrium for a month at a dose of 64 Gy in 16 Fr. There was no local recurrence 6 months after surgery. However, metastasis to the lungs was identified. Although the patient received chemotherapy (paclitaxel and cisplatin), lung and liver metastases progressed (Fig. 4). Unfortunately, the patient died of multiorgan failure due to multiple metastases 17 and 18 months after surgery and diagnosis, respectively.

## 3 Discussion

Primary cardiac tumors have an incidence of 0.001–0.03 % on autopsies, and are benign in approximately 75 % of cases. Among the remaining 25 % of tumors, sarcomas are the most common [3]. Angiosarcomas usually originate in the right atrium and frequently present with nonspecific symptoms, such as dyspnea, cough, heart failure, and arrhythmia. Delayed diagnosis is associated with a poor prognosis [4].

Imaging studies are essential for diagnosing these tumors. They aid in determining tumor size, location, and relationship to the pericardium or valves. These tumors appear hyperintense or isodense to the myocardium on T1-weighted or proton density-weighted MRI. On T2-weighted imaging, they appear hyperintense due to increased vascular flow [5]. In our case, the tumor showed a high-intensity signal on T2-weighed MRI. Pericardial fluid cytology is unreliable and should not be performed. Malignant cells are rarely found in the sanguineous fluid, even when the tumor has invaded the pericardium [6]. However, the final diagnosis is often made following thoracotomy or autopsy. Transvenous biopsy has several disadvantages, including the risk of bleeding from a highly vascularized mass and induction of metastatic spread [1]. Therefore, we performed surgery without a biopsy for the final diagnosis.

Due to the rarity of cardiac angiosarcomas and the lack of literature, there are currently no established guidelines for its treatment. However, multidisciplinary therapies, including surgery, radiotherapy, and chemotherapy, are most commonly employed. If the tumor is localized, surgical resection is the mainstay treatment and is useful for the final diagnosis. Unfortunately, many patients are in the advanced stages of angiosarcoma by the time of diagnosis, and the tumors are typically too large to be amenable to resection. The median survival for those who underwent complete surgical excision was 17 months, whereas it was 6 months for those who could not achieve complete remission [7]. Furthermore, patients with angiosarcoma had a shorter survival of 5 months than patients with other histological sarcomas [7]. This shows that cardiac angiosarcoma continues to have a poor prognosis.

The use of adjuvant therapies, including chemotherapy and radiotherapy, is debatable. No randomized trials have been performed to provide evidence for these adjuvant therapies. Furthermore, since cardiac angiosarcomas are rare, most available treatment modalities are for extra-cardiac angiosarcomas. The traditional cytotoxic chemotherapy drugs for angiosarcoma are anthracycline, ifosfamide, and taxanes (paclitaxel and docetaxel) [8]. Adjuvant chemotherapy with a doxorubicin-containing regimen did not benefit patients with primary cardiac sarcomas, including angiosarcomas [9]. A clinical trial of first-line anthracycline-based chemotherapy for angiosarcoma demonstrated a median progression-free survival of 4.8 months and an overall survival of 9.9 months, which are not significantly different from those of other soft tissue sarcomas [10]. The role of chemotherapy in metastatic settings is unclear. However, successful treatment of some cases of cardiac angiosarcoma has been reported. A combination of docetaxel and radiotherapy maintained a progression-free survival of 12 and 16 months in two patients [11], [12]. Another patient who received radiochemotherapy after surgery survived for more than 3 years after treatment but later died of liver metastasis, although local recurrence was absent [13]. In our case, local recurrence was absent, and pericardial effusion did not appear after surgery. The 18 months of survival after diagnosis in our report is longer than that observed in other reports. Hence, the combination of surgery and radiochemotherapy (with heavy ions) was useful.

## 4 Conclusion

Herein, we present a case of primary cardiac angiosarcoma that was managed with surgical resection and chemoradiotherapy. Cardiac angiosarcomas are rare and difficult to diagnose in advance because they are associated with few symptoms. They have no standard treatment; however, surgical resection, chemotherapy, radiotherapy, or a combination of these therapies may be useful.

## Sources of funding

This research did not receive any specific grant from funding agencies in the public, commercial, or not-for-profit sectors.

## Ethical approval

This study was granted an exemption from full ethical review, since clinical pictures of the patient or any information that could directly identify the patient were not used.

Data are available upon request.

## Consent

Written informed consent was obtained from the patient's family for publication of this case report and accompanying images. A copy of the written consent is available for review by the Editor-in-Chief of this journal on request.

## Author contributions

A.K., T.T., and O.K. performed the surgery and patient care postoperatively. S.T. managed the patient for chemotherapy and radiotherapy. The first draft of the manuscript was written by A.K. All authors have read and approved the final manuscript.

## Research registration

This case report is not “First in Man” study.

## Guarantor

Akihiro Kobayashi.

## Provenance and peer review

Not commissioned, externally peer-reviewed.

## Declaration of competing interest

The authors declare that there is no conflict of interest regarding the publication of this article.
